# Retinoic Acid, Leaky Gut, and Autoimmune Diseases

**DOI:** 10.3390/nu10081016

**Published:** 2018-08-03

**Authors:** Leila Abdelhamid, Xin M. Luo

**Affiliations:** Department of Biomedical Sciences and Pathobiology, College of Veterinary Medicine, Virginia Tech, Blacksburg, VA 24061, USA; leila88@vt.edu

**Keywords:** retinoic acid, leaky gut, autoimmune diseases

## Abstract

A leaky gut has been observed in a number of autoimmune diseases including type 1 diabetes, multiple sclerosis, inflammatory bowel disease, and systemic lupus erythematosus. Previous studies from our laboratory have shown that lupus mice also bear a leaky gut and that the intestinal barrier function can be enhanced by gut colonization of probiotics such as *Lactobacillus* spp*.* Retinoic acid (RA) can increase the relative abundance of *Lactobacillus* spp. in the gut. Interestingly, RA has also been shown to strengthen the barrier function of epithelial cells in vitro and in the absence of probiotic bacteria. These reports bring up an interesting question of whether RA exerts protective effects on the intestinal barrier directly or through regulating the microbiota colonization. In this review, we will discuss the roles of RA in immunomodulation, recent literature on the involvement of a leaky gut in different autoimmune diseases, and how RA shapes the outcomes of these diseases.

## 1. Retinoic Acid and Its Signaling

Retinoic acid (RA) is a multifunctional metabolite of vitamin A that has been known as the maestro behind various functions of vitamin A [1]. RA has three natural isomers including 9-*cis*, 13-*cis*, and all-*trans* RA that have shown different capacities for modulating cellular differentiation and proliferation [2,3,4]. The different abilities of these isomers to regulate cellular growth and differentiation could be attributed to their different affinities to their nuclear receptors, namely, RA receptors (RARs) and retinoid X receptors (RXRs), that in turn derive selective functionalities as previously reviewed [4]. For instance, all-*trans* RA binds only to RARs and can induce RXR activation indirectly though isomerization with 9-*cis* RA, which possesses the affinity to both RARs and RXRs [5].

All-*trans* RA (hereafter referred to as RA) has been recognized for its immunomodulatory capacities [6,7], but many functional controversies exist where the mechanisms of RA actions remain uncovered. The advancement of scientific technologies such as cloning, sequencing, and proteomic analysis has unraveled the molecular mechanisms behind RA-induced immunomodulation. Knowledge relating to the discovery of retinoid receptors and the rising themes on their mechanisms of action have been reviewed elsewhere [8]. However, how the binding of RA with its receptors may induce different functions is still an active area of research. Here, we briefly review RA signaling through binding to different RAR isotypes (RARα, β, γ) that work selectively via heterodimerization with RXRs [9]. RXRs are known to heterodimerize with other nuclear receptors such as vitamin D receptors [10]. Binding of RA to RAR subtypes induces the formation of heterodimer complexes with RXRs and subsequently regulates various biological activities and cellular fate decisions [11]. Generally, nuclear hormone receptor ligands like RA could modulate cellular gene expression through direct modulation of transcription via binding to specific genes promoters and/or indirectly though nongenomic extranuclear pathways [12]. RAR–RXR dimerization directly induces their binding to specific DNA sequences known as RA response elements (RARE) [13], subsequently promoting and regulating a complex niche of genes that induce differentiation of cells into functionally distinct phenotypes [8]. 

Activation of different RAR isoforms initiates variable and even paradoxical outcomes in different cellular contexts [14,15]. Indeed, along with other factors, including cellular growth factors and cytokine regulation in different cell types, a strictly regulated RA–RAR interaction is needed for sustaining homeostasis. Dysregulation of such integrity can shift from a steady state to diseases [16]. For instance, the ability of RA to induce neutrophil lineage differentiation from common hematopoietic progenitors is controlled by its ligation to RARα. Consequently, the perturbation in RA–RARα ligation has been proposed to contribute to neutrophil-associated leukemic phenotypes [17,18]. In addition, the ligation of RA to different nuclear receptors controls both cellular apoptotic and survival signals. For instance, the shifting of RA from binding to the ordinary RARs to binding to the orphan PPARβ/δ receptors hinders the ability of RA to control cellular growth and increases the expression of prosurvival genes leading to cellular hyperplasia [14]. Therefore, the functions of RA differ by ligating to distinct RAR variants as well as by the interaction of RARs with different coregulators (coactivators or corepressors) [19]. 

In addition to direct genomic transcriptional regulation, RA can initiate the crosstalk between genomic- and nongenomic-driven cellular modulation [20]. Through non-transcriptional mechanisms, RA–RAR ligation regulates multiple extranuclear activation cascades, including mitogen-activated protein kinase (MAPKs) and others. The RA-mediated activation of different kinase cascades is driven by phosphorylation of RAR subtypes. This initiates kinase integration into the cell nucleus, allowing their binding to specific gene promoters and subsequently modulating cell differentiation [21]. Through this mechanism, RA is involved in the regulation of different kinases affecting the cell cycle machinery and the functional differentiation of normal immune cells, including B lymphocytes [22,23], T lymphocytes [24], and natural killer-T (NKT) cells [25]. Therefore, the immunomodulatory capacities of RA to regulate cellular fates and functions is strictly regulated by RA–RAR signaling and dependent on the microenvironment. 

Indeed, the interplay between RARs, cytokines, and kinases can explain some mysteries that regulate cellular fates and functions. For example, RARγ has been recently found to mediate cellular life-to-death switch signals by modulating the tumor necrosis factor (TNF)-induced inflammatory versus apoptotic shifts [26]. The release of RARγ into the cytoplasm in the absence of cellular inhibitor of apoptosis (cIAP) induces the dissociation of receptor-interacting protein kinase 1 (RIP1) from the TNF receptor and subsequently initiates the formation of cytoplasmic death complexes, therefore shifting from TNF-induced inflammatory signals to TNF–RIP1-induced apoptosis [26]. 

Furthermore, previous reviews have shown the roles of RA as a pleiotropic modulator of immune cells to maintain homeostasis especially in intestinal compartments [7]. For instance, how RA–RAR signaling affects both immunogenic and/or tolerogenic responses of different immune cells including antigen presenting cells (APCs, such as dendritic cells (DCs)) and the T helper-17 (Th17)-T regulatory (Treg) cell axis has been previously discussed [27]. This depends not only on the type of RAR isoforms but also the dose of RA and the affected cell types and can trigger different and sometimes unpredictable outcomes. Similarly, Czarnewski and colleagues recently reviewed the RA-induced modulation of intestinal innate cells, including DCs and innate lymphoid cells (ILCs) [28].

Therefore, in this review, we further expand the discussion on how RA and RARs influence different immune cells, including B lymphocytes, T lymphocytes, and myeloid cells, and how this modulation could vary depending on the cellular developmental stage and the milieu.

## 2. Roles of RA on Immunity

RA effectively contributes to immunity through its intricate crosstalk with different immune elements. RA signaling drives different cell lineage decisions as well as the function of effector immune cells in different contexts.

### 2.1. RA and B Lymphocytes

B lymphocytes are important regulators of the immune system, for which their effector functions include immunoglobulin (Ig) production for antigen (Ag) neutralization, control of optimal immunization responses, and Ag recognition and presentation to stimulate other effector cells. B-cell dysregulation has been linked to various autoimmune inflammatory conditions [29], and many reports have shown their potent regulation of autoimmunity [30,31].

Different studies have investigated the effect of RA on B-cell development, maturation, and functions. RA selectively promotes B-cell lymphopoiesis in the bone marrow (BM) through RARγ signaling in Nestin (a regulator of lymphopoiesis)-expressing hematopoietic cells [32]. In addition, RA is proposed to create a microenvironment that encourages functional differentiation of B cells [33,34]. For instance, the induction of regulatory immunosuppressive B cells (Breg cells) that have protective capacities under many autoimmune conditions largely depends on RA. The ability of tolerogenic DCs to induce CD19^+^CD24^+^CD38^+^ Breg cells depends on production of RA from DCs and the expression of RARs in the CD19^+^CD24^+^CD38^+^ population. In vitro treatment of B cell–DC cocultures with ER50891, a pan-RAR antagonist, hindered the induction of Breg cells [35]. This finding indicates the significance of RA responsiveness for the immune regulation directed by B lymphocytes. 

The crosstalk between RA and B cells is necessary for the optimal adaptive responses of B cells in maintaining immune homeostasis. RA has been found to be critical for the humoral T-independent responses of B-cells. Altered levels of IgM and IgG3 and reduction of the functionally developed antibody-secreting cells have been observed with the silencing of RARα expression in a murine model overexpressing dominant-negative RARα (dnRARα) [36]. Similarly, RARα signaling is essential for the isotype switching of antigen-specific B cells to IgA-secreting cells. Loss of RA responsiveness in the CD19^Cre^-dnRARα model has been linked to reduced IgA-secreting cells both in vitro and in vivo. Additionally, RARα-silenced cells exhibit reduced expression of the gut-homing integrin α4β7, indicating the effect of RA on B-cell migration as well [37].

However, RA affects the isotype switching and homing capacities of only certain subsets of B lymphocytes. At steady states, RA signaling paradoxically promotes IgA-secreting cells with no similar responses on IgG- or IgM-secreting cells. For instance, RARα agonists can increase IgA levels that are diminished by RARα antagonist-induced blockade; however, neither the agonist nor the antagonist of RARα affects the production of other Ig isotypes [38]. Similarly, the intrinsic propensity of RA in modulating the B-cell response is not equal towards all B-cell subsets; instead, the homing and secretory functions of some B cells are more prominently affected. RA has been found to mostly influence the IgA and α4β7 expression capacities of murine peritoneal B1b cells rather than other peritoneal or splenic B cell populations such as B1a and B2 cells [39]. These findings have been confirmed by different reports that tested RA-induced class switching of B cells in the presence of an activator for B2 cells, such as the mucosal glycoprotein lactoferrin (LF). LF treatment is reported to influence IgA and IgG production by B1 and B2 cells, respectively [40]. Interestingly, even with LF–RA combination treatment, the IgA class-switching and the homing capacities—as indicated by enhanced α4β7 and chemokine receptor CCR9 expression—were only restricted to B1 cells [41], confirming the paradoxically selective action of RA on certain B-cell subsets. 

However, in contrast to the RA-mediated preference of B-cell switching to IgA-producing cells, RA can also mediate polyclonal differentiation of B lymphocytes upon sensing the microenvironment under pathogenic conditions through triggering toll-like receptor 9 (TLR9) and CD180 antigen (TLR homolog leucine-rich repeat 105) [42]. RA has been shown to normalize the proliferation of defective B cells and restore their IgG-producing capacities as well as IL-10 production in the presence of TLR9/CD180 stimulation in common variable immunodeficiency (CVID) models [43]. RA is also known to be able to induce interferon regulatory factor 4 (IRF4) and Blimp1 (a repressor of IFNβ gene expression); both are plasma cell-generating transcription factors that can promote somatic gene recombination in the variable and conserved regions of the Ig molecule. This results in class switching with subsequent IgG production in TLR9/CD180-activated human B cells [44]. 

Furthermore, the effects of RA on B-cell responses not only depend on the health conditions (steady state versus pathologic state) but are also cell- and tissue-dependent. For instance, unlike the effect of RA on the gut-homing integrin α4β7 expression in murine proteneal B1 cells, RA does not influence this integrin expression in other B cell subsets in other tissues such as the splenic marginal zone (MZ) B cells. In contrast, the blockade of RARα signaling in the dnRARα model as previously discussed drastically reduced the homing receptor Sphingosine-1-phosphate receptor-1 (S1P1) expression on MZ B cells with no effect on integrin α4β7 expression [36]. These data further highlight the complex interplay between RA and B cells to induce immune regulation in normal versus sensitized conditions in a tissue- and cell-specific manner. 

### 2.2. RA and T Lymphocytes

It is well established that RA is essential in maintaining gut-tropic regulatory T (Treg) cell responses and the balance between immunity and oral tolerance [45]. RA reciprocally regulates T helper 17 (Th17) and Treg cells to maintain immune homeostasis [7]. Under normal conditions, RA favors Treg cells over Th1/Th17 differentiation and controls the differentiation [46,47], the homing, and the functional capacities of Treg cells [48,49].

The interplay between RA and different mediators, such as transforming growth factor β (TGFβ) [50], interleukin 2 (IL-2) [51], and IL-1 family of inflammatory cytokines [52,53], could drive the Treg–Th17 balance. Here, we will discuss the RA-induced regulation of different T-cell subsets, including CD4^+^, CD8^+^, and γδ T cells, meditated by RAR signaling.

Interestingly, as for B cells, the effect of RA on T lymphopoiesis depends selectively on RARγ signaling. Whether RARγ mediates thymic single positive (SP) CD4^+^ or CD8^+^ T cells’ differentiation lineage specificity is unclear. However, knocking down RARγ in Nestin-expressing thymic cells reduces the thymic double negative (DN) T-cell precursors, as well as CD4^+^ T cells, immature SP CD8^+^ cells, and CD4^+^CD8^+^ double positive (DP) T cells in the peripheral circulation [32]. Interestingly, RA treatment could reverse the effects of RARγ depletion. Thus, the crosstalk between RA and the thymic microenvironment may govern CD4^+^ or CD8^+^ T-cell differentiation capabilities. 

Among different RAR isoforms, RARα is proposed to be the most important for RA-directed modulation of T-cell responses. Even though no prominent difference in CD4^+^ T-cell numbers was observed with deletion of different RAR isoforms, RARα was shown to be essential for CD4^+^ T-cell immunity following both oral infection and immunization [54]. Specific deletion of RARα in CD4^+^ T cells drastically reduced their interferon-γ (IFNγ) production and was comparable to vitamin A deficiency (VAD)-induced disruption of T-cell homeostasis (e.g., Treg-Th1/Th17 imbalance) [54]. However, RA promotes either inflammatory (Th1) or tolerogenic (Th2) CD4^+^ T-cell lineages in different contexts. For example, concomitantly with tetanus toxoid (TT) immunization, RA treatment dampened IFNγ and IL-12 production and increased IL-4/ IFNγ ratios in C57BL/6 mice, indicating that RA mediated Th2 over Th1 responses [55]. RARα signaling also promoted Th2 differentiation under inflammatory conditions. For instance, in trinitrobenzene sulfonic acid (TNBS)-induced murine colitis, RARα agonist injection skewed the lamina propria CD4^+^ T cells toward a Th2 phenotype. RA treatment in this mouse model decreased IFNγ, IL-2, and TNF production while favoring the production of Th2 cytokines, including IL-4 and IL-10 [56]. On the contrary, in a T-cell transfer colitis model, RA was required for the induction of CD4^+^ T-cell differentiation and expansion into the Th1 lineage. Blockade of RARα responsiveness skewed CD4^+^ T-cell differentiation toward IL-17^+^ and IL-17^+^Foxp3^+^ T cells and greatly diminished their capacity to differentiate into IFNγ-producing Th1 cells, thus collectively interfering with the generation of intestinal inflammation [57]. Additionally, RARα silencing in the same model has been associated with the inability of colonic CD4^+^ intraepithelial lymphocytes (IELs) to differentiate into cytotoxic T lymphocyte (CTL)-like cells [58]. Those findings indicate variable functions of RA–RAR signaling in CD4^+^ T cells under different contexts.

RAR signaling is similarly significant for CD8^+^ T-cell responses. Similar to its effect on CD4^+^ T cells, RARα has a potent effect on other RAR isoforms in modulating CD8^+^ T-cell responses following infection. Even though the absence of different RARs did not affect the initial T-cell activation, RARα was essential for CD8^+^ T-cell survival and their effector functions. The conditional deletion of RARα in CD8^+^ T cells reduced IL-2 and IFNγ production, gut-homing propensity (α4β7 and CCR9 expression), and Ag-specific clonal expansion following *Listeria monocytogenes* infection [59].

Interestingly, RA-mediated immune homeostasis is not confined to the adaptive compartments. RA can also regulate the interface between innate and adaptive responses. This is done through mediating different functions of γδ T cells, a less abundant T-cell population that is mostly found within IELs and links between both arms of the immune system [60]. Little is known about the molecular mechanisms on how RA modulates γδ T-cell responses. However, RA has been reported to diminish the production of inflammatory cytokine IL-17A from γδ T cells and further their production of the anti-inflammatory cytokine IL-22 [61]. Therefore, RA indirectly diminishes the Th17 response that is a major contributor of autoimmunity, as IL-17A production from γδ T cells has been proposed to augment the Th17 response [62]. Moreover, γδ T cells have recently been shown to play major roles in initiating excessive autoimmune inflammation [63,64,65]. Therefore, the effect of RA on γδ T cells suggests a promising strategy to attenuate autoimmune conditions such as the central nervous system (CNS) autoimmunity by suppressing IL-17 production [66]. Further studies on the molecular mechanisms behind RA-mediated modulation of γδ T cells are warranted and may provide insights and targets for controlling hyper-inflammatory signals. 

### 2.3. RA and Myeloid Cells

RA is well established as a pluripotent hematopoietic regulator [67,68]. The hematopoietic regulatory capacities of RA are driven through the interaction between RA and its nuclear receptors RARα and RARγ. RARα bidirectionally controls granulocytopoiesis by promoting cell differentiation in the presence of RA but inhibiting it in the absence of RA [69]. On the other hand, RARγ hinders hematopoietic stem cell (HSC) differentiation in favor of their self-renewal and proliferation [70]. Recent findings suggest that the expression and activities of both RARs coordinate to derive the differentiation lineages of HSCs, and that this is largely dependent on the bioavailability of RA [71]. Therefore, different doses of RA can determine different cellular lineage specificities.

The dose of RA can determine different myeloid cell (MC) differentiation or functional outcomes as well as different myeloid specificities. For instance, during early monopoiesis, continuous RA treatment promotes the differentiation of common bone marrow progenitor cells (BMPCs) into regulatory MCs. In vitro supplementation of RA in BMPCs in the presence of granulocyte–monocyte colony-stimulating factor (GM-CSF) increases the proliferation of mature regulatory CD11b^+^CD11c^-^Ly6C^low/intermediate^ monocytes. This is indicated by enhanced expression of both maturation markers, including CD80, CD86, and MHC-II, as well as the suppressive/inhibitory markers including PD-L1 and PD-L2. On the contrary, different responses were observed for mature CD11c^+^ DCs in the same study, suggesting that the influence of RA varies depending on different developmental stages of MC differentiation [72]. Moreover, RA has been found to improve the differentiation of myeloid-derived suppressive cells (MDSCs), a heterogenous immature population that possesses a significant ability to suppress both innate and adaptive responses through direct modulation of cytotoxic T cells (CTLs) and natural killer (NK) cells [73]. The effect of RA on these cells enhanced the antigen-specific responses in cancer patients [74] in a time-dependent fashion [75]. Together, these findings suggest that during early granulocytopoiesis, RA may enhance the immature MCs to express immunosuppressive/regulatory profiles.

In addition, RA promotes the anti-inflammatory capacities of mature monocytes, consequently dampening tissue inflammation. For instance, RA regulates the TLR sensing of monocytes, where RA treatment of primary human monocytes was associated with downregulated TLR2 expression and reduced TLR2- and TLR4-mediated inflammatory cytokine production [76]. Similarly, for in vitro differentiated human macrophages, RA treatment promoted anti-inflammatory responses, indicated by enhanced GM-CSF, monocyte chemotactic protein-1 (MCP-1), and IL-10 production, while reducing IL-6, macrophage inflammatory proteins (MIP)-1α, and MIP-1β following lipopolysaccharide (LPS) stimulation [77]. Furthermore, in an in vitro *Bacillus Calmette–Guérin* (BCG)-primed human monocyte model, RA inhibited the production of cytokines from stimulated monocytes and diminished their response to microbial restimulation. Mechanistically, RA promoted the histone inhibitory signature H3K9me3 and inhibited the stimulatory signature H3K4me3, thereby downregulating the cytokine response of monocytes upon restimulation [78]. RA is proposed to modulate histone methylation through regulating the expression of methyl transferase SUV39H2. On the contrary, for tissue macrophages, the effect of RA may vary according to their tissue microenvironment (e.g., intestinal versus extraintestinal tissues). For example, silencing of RAR signaling in intestinal macrophages isolated from Crohn’s disease patients has been associated with a reduced inflammatory macrophage profile—decreased CD14 and human leukocyte Ag (HLA)-DR expression as well as decreased TNFα production—indicating that RA responsiveness is essential for TNFα-induced inflammatory macrophage responses [79]. Therefore, the uptake of RA and/or its intrinsic production by intestinal macrophages may contribute to the macrophages’ role in Crohn’s disease pathogenesis [79]. This finding suggests that RA may worsen intestinal inflammation during progressive stages of inflammation through potentiating the macrophages’ pro-inflammatory response. In contrast, in an extraintestinal environment, as in the alcoholic liver disease (ALD) model, reduced RA concentration in vivo was correlated with aggravated disease pathogenesis through priming of peritoneal macrophage-mediated inflammatory responses. Consistent with this, RA supplementation decreased the TNFα- and nitric oxide (NO)-induced pro-inflammatory state in peritoneal macrophages [80]. These findings suggest that the RA-mediated regulation of the differentiation and functions of MCs varies depending on cellular developmental stages as well as their surrounding microenvironment under normal versus diseased conditions. This calls for further studies on the mechanisms and influencing factors regulating the interaction between RA and MCs. 

## 3. Efficacies of RA in the Treatment of Autoimmune Diseases

**Figure 1:**
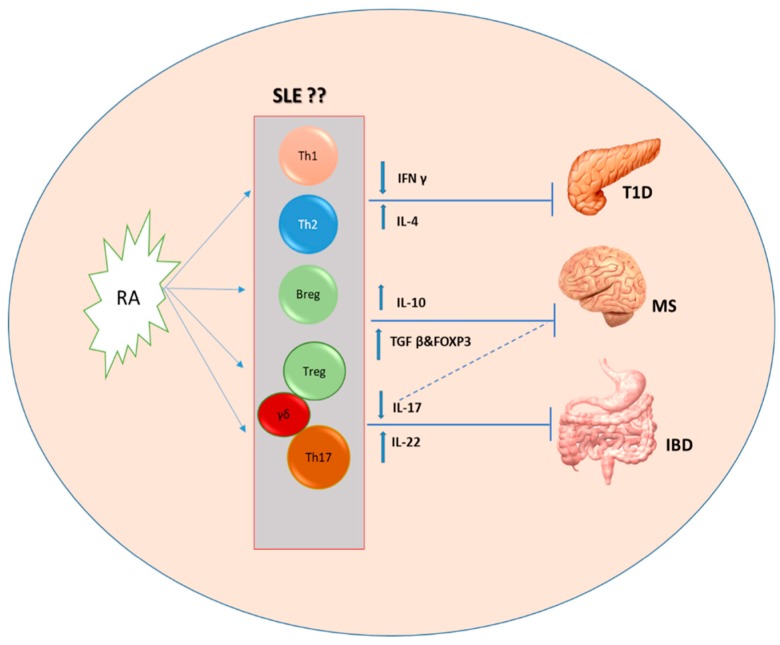
Retinoic acid (RA)-mediated cellular regulation in autoimmune diseases. RA suppresses the inflammatory T helper (Th)1/Th17 responses by decreasing interferon-γ (IFNγ) and interleukin (IL)-17. It also primes a regulatory/anti-inflammatory environment by enhancing IL-4, IL-10, IL-22, and transforming growth factor β (TGFβ). Through these mechanisms, RA may dampen different pathologies, including type 1 diabetes (T1D), multiple sclerosis (MS), and inflammatory bowel disease (IBD). However, the cellular mechanisms of RA in modulating systemic lupus erythematosus (SLE) are yet to be uncovered.

Autoimmune diseases are idiopathic multifactorial conditions that have been characterized by the breakdown of self-tolerance. In such cases, the immune system deviates from attacking nonself-invaders to attacking self-tissues, resulting in multiple tissue damages [81]. The roles of RA on autoimmunity could be bidirectional depending on different factors, including the tissue microenvironment and the stage of disease. For instance, supplementation of RA can ameliorate some tissues’ pathologies, as in the kidney during lupus nephritis, while deteriorating other tissue pathologies, for instance, the lung and skin lesions [82]. Similarly, RA treatment exacerbates neuroinflammation by increasing autoantibodies, total IgG, and complement C3 protein deposition in the brain tissue and has been associated with more severe neurodegeneration in MRL/lpr mice [83]. Furthermore, with its potential toxicity [84,85], employing RA as a potential therapeutic for autoimmune disorders requires a deeper consideration for the context in which RA interplays. Further research on developing fine-tuned RA-mediated therapies that consider the different RA signaling pathways is necessary to avoid any undesired outcomes, especially for long-term therapies. Here, we highlight efficacies of RA in the treatment of different autoimmune diseases (Figure 1).

### 3.1. Type 1 Diabetes

Type 1 diabetes (T1D), or juvenile diabetes, is an idiopathic condition in which pancreatic islets are destroyed through the autoimmune-mediated destruction of β cells that hinders their ability to produce insulin, thus resulting in high blood glucose levels [86]. Ongoing research findings reveal a protective effect of RA against T1D in mice. For instance, RA prevented the onset of T1D even after the establishment of insulitis by suppressing the IFNγ-producing T cells, upregulating Treg proliferation, and hindering the CD8^+^ T-cell infiltration into pancreatic islets, consequently reversing their cytotoxic activities in the nonobese diabetic (NOD) mouse model as well as the adoptive transfer model in NOD/*scid* mice [87]. In addition, RAR signaling is essential for continued β-cell responsiveness to glucose and sustaining their insulin production [88]. RA treatment has been shown to interfere with T1D progression and results in the maintenance of blood glucose in Streptozotocin (STZ)-induced T1D. RA supplementation to this mouse model inhibited STZ-induced β-cell damage, consequently improving the serum insulin levels and enhancing glucose uptake, thus lowering the blood glucose levels. Immunologically, RA reduced the inflammatory IFNγ and increased the anti-inflammatory IL-4 levels [89]. Subsequently, RA treatment was able to lessen pancreatic destruction through counteracting with the inflammatory niche-induced damage. However, after the initiation of T1D, RA treatment might not be sufficient to reverse hyperglycemia nor improve the survival rates even when combined with exendin-4, a glucagon-like peptide-1 receptor agonist that enhances pancreatic β-cell function [90]. These findings indicate that disease stage-specific modulation of the immune system with RA and RA therapies could be promising preventions but not cures for T1D. 

### 3.2. Multiple Sclerosis

Multiple sclerosis (MS) is an autoimmune chronic neuroinflammation that affects the CNS [91]. MS could be a consequence of perturbed microbiota that creates a peripheral inflammatory milieu promoting disease [92,93]. The efficacies of different RA isomers in modulation of MS and its experimental animal model, experimental autoimmune encephalitis (EAE), have been previously reviewed [94]. Here, we further expand on the discussion. In the neuroinflammatory context of MS, RA showed various beneficial effects [95]. In clinical trials, supplementation of RA was not correlated with MS exacerbation [96]. On the contrary, RA enhanced the overall quantitative assessment or MS functional composite of relapsing–remitting MS patients [97]. Moreover, RA treatment reduced fatigue and depression symptoms that often occur with relapsing cases of MS [98].

Indeed, vitamin A has been proposed to derive intrinsic immunoregulation within the CNS tissue under the MS condition [99]. RA is intrinsically produced by the reactive astrocytes that exhibit upregulated RALDH2 expression. This endogenously produced RA is linked to improved blood–brain barrier (BBB) function and diminished inflammatory responses as represented by reduced monocyte adhesion and activation in the brain [100]. 

Additionally, RA has been found to ameliorate the development of MS through improving the humoral and cellular responses. RA modulates B-cell responses [101] and re-establishes the inflammatory versus immunoprotective T-cell balance [102] in MS patients. For instance, RA treatment initiated comparable responses from B cells similar to those obtained with the first line drugs widely used for MS, including glatiramer acetate (GA) and IFNβ-1b. RA enhanced IL-10 production from TLR-stimulated B cells isolated from RA-treated relapsing MS patients without affecting the inflammatory TNFα [101]. In addition, RA upregulated Foxp3 and TGFβ gene expression in PBMCs isolated from clinically treated patients, indicating skewed T-cell populations toward Treg responses [102]. Moreover, in the experimental MS model, RA treatment hindered IL-17 production from γδ T cells and reduced their infiltration into the CNS. In fact, RA supplementation to γδ T cells was correlated with their mitigated ability for disease induction [66]. These findings highlight the potential role of RA in halting MS initiation and progression.

### 3.3. Inflammatory Bowel Disease

Inflammatory bowel disease (IBD) is one of most prevalent autoimmune diseases in the Western and industrialized world [103], where the epidemiological data correlates the Western lifestyle to disease pathogenesis [104]. Indeed, IBD is a multifactorial cycle of mucosal damage and breaching of the balance between luminal antigens and mucosal immunity [105,106]. 

Regulation of intestinal homeostasis through RA [7] may encourage the utilization of RA-based therapies for controlling the breakdown of intestinal tolerance that could lead to IBD progression [106]. RA may mitigate IBD severity through various immunoregulatory mechanisms. RA could: (1) restore and/or reprogram the impaired Treg/Th17 lineage differentiation that is usually linked to IBD development [51,107] by inducing adaptive Treg cells and imprinting their gut-homing phenotype in response to inflammatory stimuli [108]; (2) modulate the recognition of different TLR ligands and control the activation of downstream transcription factor signaling [109,110]; (3) downregulate inflammatory signaling molecules like nitric oxide (NO) from PBMCs of IBD patients, even after the establishment of the pro-inflammatory niche [111]; (4) regulate the production of immunoregulatory cytokines, such as by enhancing the synthesis of IL-22 by γδ T cells and ILCs and consequently attenuating colitis [61]; and (5) synergize with immunoregulatory mediators like TGFβ for maintaining gut homeostasis [112].

Interestingly, RA has been found to not only mitigate intestinal inflammation but also counteract IBD-related consequences such as necrotizing colitis and tumorigenesis. Altered metabolism of RA was correlated with increased tumor burdens in ulcerative colitis (UC) patients due to inhibition of tumor-clearing CD8^+^ CTLs, whereas RA normalization induced protective CD8^+^ T-cell activities [113]. In addition, blockade of CYP26A1 that restores RA levels in experimental murine IBD models could diminish both intestinal inflammation and tumor development [114]. Moreover, RA treatment has been found to downregulate TNFα and NO synthase 2 (NOS2) levels that are associated with UC and colitis-associated cancer (CAC). Mechanistically, RA was able to dampen TLR4/NF-κB signaling, and this highlights new targets for tackling UC and CAC [115]. Therefore, employing the ability of RA to modulate mucosal immunity may help in controlling IBD and its pathological consequences.

### 3.4. Systemic Lupus Erythematosus

Systemic lupus erythematosus (SLE), or lupus, is a multiorgan autoimmune-driven destruction [116,117]. As a natural metabolite, RA could provide a less risky treatment for SLE, especially when compared to the severe adverse effects of the immunosuppressants such as corticosteroids and cyclophosphamide [118,119]. RA treatment has been shown to diminish the severity of lupus nephritis, one of the most prevalent manifestations to SLE in both animal models [120] and clinical trials of lupus patients [119]. 

Studies have shown various mechanisms through which RA treatment may modulate the course of SLE. For example, RA has been reported to restore the downregulated lactobacilli—a key feature of leaky gut in MRL/lpr mice—thus ameliorating the inflammatory symptoms of lupus [121,122]. In addition, supplementation of RA that restored vitamin A hypovitaminosis in SLE patients re-established the Th17/Treg balance that is often skewed for lupus induction [123]. Moreover, RA interferes with the signaling of transcriptional regulators needed for lupus initiation. For example, RA has been found to inhibit the activities of prolyl isomerase Pin1, a regulator of interleukin-1 receptor-associated kinase 1 (IRAK1) in the TLR7/TLR9 signaling pathway [124]. Similarly, RA can inhibit interferon regulatory factor 7 (IRF-7) signaling, which is associated with deteriorated lupus. Furthermore, in vitro RA supplementation to PBMCs derived from lupus patients was able to suppress IRAK1/IRF-7 signaling [124]. Therefore, RA control of Pin1 activation was able to significantly inhibit lupus initiation and improve the overall phenotypic parameters in both MRL/lpr and B6/lpr lupus-prone mice [124]. Dissecting the underlying mechanisms of action for RA may help the development of more efficient and less risky targets to inhibit lupus initiation in genetically predisposed individuals. 

## 4. The Leaky Gut and Autoimmune Diseases

Even though the exact etiology of autoimmune conditions is vague, a leaky gut has been associated with various autoimmune diseases including T1D [125,126], MS [127,128], IBD [129], and SLE [130]. A previous review by Arrieta et al. (2006) discussed the hypothesis that impaired intestinal permeability and the breakdown of barrier functions are correlated with either local or systemic autoimmune inflammation. Experimental evidence was provided to support this hypothesis [131]. Studies from our laboratory have shown the presence of a leaky gut with impairment of intestinal barrier functions in the genetically predisposed lupus-prone MRL/lpr mice [122]. Here, we will discuss the regulation of intestinal barrier function and the potential mechanisms of how a leaky gut can contribute to autoimmune diseases.

### 4.1. Regulation of Intestinal Barrier Function

The intestinal mucosa has a unique physiology that makes it a system of its own. Despite exposure to a tremendous number of external antigens from the food that we consume, the intestinal mucosa tightly regulates the balance between tolerance and immunity. This can be achieved through a complex loop of interaction between the physical and immunological defenses of the gut [132]. The coordination between these defenses can regulate the production of important immune modulators that are necessary for gut immune homeostasis, e.g., the production of secretory IgA that plays a major role in gut immunity [133].

The intestinal mucosa is a strong physical barrier with a monolayer of intestinal epithelial cells (IECs) interconnected by a network of intercellular junction proteins known as tight junctions (TJs). It keeps the spatial segregation between gut microbiome and food-derived antigens in the intestinal lumen and the underlying lamina propria tissues where the immunological barriers exist [134]. This physical defense is achieved through the inner intestinal wall and its mucus coverage. The secretion of mucin by goblet cells forms a protective barrier that traps pathogens and prevents their invasive colonization [135]. Therefore, the breakdown or dysregulation of mucin is often associated with intestinal inflammation [136], and the integrity of the mucus layer helps to maintain a healthy mucosal functionality [137].

The intestinal mucosa is an important regulator of host immunity. For instance, the intestinal mucosa is enriched with different antimicrobial peptides that are secreted from Paneth cells within the intestinal epithelial lining. Such antimicrobial peptides, including cathelicidins and defensins, can regulate the intestinal barrier function and mediate immune defenses against bacterial invasion [138]. Furthermore, IECs themselves are known for their ability to regulate the proliferation and functional differentiation of both innate (basophils, ILCs, and macrophages) and adaptive immune cells (naïve CD4^+^ T cells and B cells). This capability of IECs to regulate the responses of different immune cells depends largely on the interaction between different cytokines produced by those cells, as previously reviewed [139].

Interestingly, IECs can also sense the microenvironment for microbial invaders and maintain immune integrity and gut homeostasis. IECs can produce both RA and TGF-β that prime tolerogenic CD103^+^ DC responses in the mucosal interface [140,141] IECs also differentially modulate the recognition signals for commensal versus pathogenic microbes. Such balance is regulated by the interplay of IECs with different components of innate defenses [142] as well as their surrounding microflora [143]. Even though IECs are in continuous contact with a myriad of microbes, they do not simply pass everything they encounter to the resident APCs in the lamina propria to initiate immunological responses. Instead, IECs have sophisticated recognition pathways where pattern recognition receptors (PRRs) on their surface can differently sense and respond to microbial stimuli according to the site of contact with the pathogens. For instance, the PRRs on IECs are either on their apical surface, which leads to tolerance, or basolateral surface, which leads to immunological responses. This distribution allows for microbial sensing by different PRRs to either inhibit or stimulate inflammatory responses. For instance, sensing of the apically distributed TLR9 suppresses the inflammatory NF-κB, whereas basolateral ones activate the NF-κB signaling. In turn, this specialized recognition can be translated into tolerance to self-microbiome versus the development of immunologic cascades against pathogens [139]. Similarly, the interaction between IECs and their surroundings controls the ability of IECs to enhance healthy functions of the intestinal barrier or provoke intestinal barrier damage with subsequent local and systemic inflammatory cascades. For example, different pathological stimuli may mediate abnormal shedding of IECs, provoking their pathological turnover, which jeopardizes the ability of these cells to maintain a healthy intestinal barrier function. Bacterial endotoxin LPS, as another example, activates excessive TNF production with subsequent TNFR1-induced NF-κB signaling, leading to excessive apoptosis of the IECs with abnormal shedding. These mechanisms initiate intestinal barrier dysfunction and can trigger hyperreactive inflammation [144]. 

Dietary mediators like RA have been recently found to alter IECs homeostasis, where depletion of RARα specifically in IECs (RARα^villin^ mice) promoted IECs differentiation into a more secretory phenotype, with increased proportions of goblet cells and Paneth cells [145]. RA is proposed to control the immunomodulatory capacities of IECs, where it could paradoxically affect IEC-directed modulation of effector lymphocytes. For instance, IECs in the presence of RA imprint T-cell polarization by either suppressing or upregulating Th17 cells in different inflammatory milieus [146]. On the contrary, they can trigger Treg proliferation under steady state [147]. Therefore, further investigation around IEC-mediated immunoregulation in different contexts may be valuable for understanding their roles in regulating the balance of the intestinal barrier.

A strict regulation of intestinal permeability is required to maintain homeostasis. This can be achieved not only through functional IECs but by the active contribution of TJs. Different integral proteins, including the claudin family, interconnect with junctional proteins such as zonula occludens (ZO) to control the transfer of different molecules through the paracellular junctions. The disruption of these packed molecules is correlated with increased permeability and the induction of intestinal inflammation [148].

A wide range of factors shape the assembly and consequently the integrity and function of TJs. These range from dietary components and the food additives in industrial products [149] to the effect of gut microbiota itself [150]. For instance, a dietary lipid-derived aldehyde called Acrolein is found to downregulate and rearrange the expression of different TJs including Claudin-1, Occludin, and ZO-1 in both in vivo and in vitro settings, which in turn affects their capability of maintaining paracellular integrity [151]. Gut microbiota and/or their products, on the other hand, influence the epithelial TJs. For example, secreted products from *Bifidobacterium infantis* were found to promote the transmembrane electrical resistance (TER) in mice treated with *B. infantis*-conditioned medium, where it enhanced the expression of ZO-1 and Occludin while decreasing Claudin-2 expression [152]. Moreover, oral treatment with *Lactobacillus* spp. such as *L. rhamnosus* and *L. acidophilus* corrected the *Shigella*-*dysenteriae*-1-induced reduction of Claudin-1 and Occludin, which in turn increased intestinal barrier function in the treated rats [153]. These findings highlight the roles of gut microbiota as well as environmental factors in shaping the epithelial TJs and subsequently the intestinal permeability.

Indeed, the assembly or disassembly of TJs depends on an intricate network of modulators that control the phosphorylation and dephosphorylation of TJ proteins. This is mainly though activation of downstream signaling of kinases (including MAPKs, protein kinase C (PKCs) and Rho kinases (RhoK)) and phosphatases, respectively. For example, targeting of integral TJ proteins such as Occludin, a main regulator for TJs assembly, by these kinases or phosphatases can directly modify the structure of other TJs and consequently their dynamic barrier functions [154].

Previous reviews have proposed the mechanisms by which pro-/anti-inflammatory cytokines disrupt or promote TJs [155,156]. Modulation of kinase activity such as phosphorylation of extracellular signal-regulated kinases (ERKs) or classical MAPKs in response to ligation of different inflammatory cytokines to their epithelial targets directly contributes to epithelial barrier dysfunction and TJ disassembly. This is indicated by increased paracellular conductance and diminished TER following different cytokine treatments [157]. For example, upregulated expression of the channel-forming TJ protein Claudin-2 was noted with IL-22 treatment of Caco-2 cells. IL-22 ligation induced the activation of JAK/STAT signaling, which is proposed to upregulate Claudin-2 protein expression [158]. This finding may explain the increased level of IL-22 with intestinal inflammation [159] and highlight the roles of cytokine-induced regulation of intestinal barrier TJs. 

Moreover, conserved microbial products such as lipopeptides and LPS were found to greatly affect the expression and continuity of TJs. For instance, sensing of TLR2 on IECs by bacterial lipopeptides has been shown to promote the phosphorylation of PKC that upregulates the expression of ZO-1 and consequently enhances the integrity of intestinal epithelium [160]. Similarly, TLR sensing upon recognition of LPS can modulate the expression of TJs and subsequently intestinal permeability [161]. Activation of TLR4/myeloid differentiation primary response 88 (MyD88) signaling through the LPS recognition by TLR4 upregulates the expression of myosin light chain kinase (MLCK) activities that can in turn modulate intestinal TJs leading to increased intestinal permeability [162]. 

Various studies have shown the direct correlation between intestinal inflammation and dysregulated junctions constituting a leaky gut [163,164]. For instance, upregulation of Claudin-2 is correlated with various IBD cases. Thus, targeting Claudin-2 through controlled intrinsic lysosomal-induced degradation or autophagy could provide a promising therapeutic intervention for such conditions [165]. Similarly, upregulation of Zonulin (a regulatory junctional protein) has been recently implicated in abnormal gut permeability and development of chronic intestinal inflammation in dextran sodium sulfate (DSS)-induced colitis [166]. Upregulated Zonulin expression was also correlated with bacterial dysbiosis and impaired permeability in clinical cases of ankylosing spondylitis [167]. These findings highlight the wide range of factors that imprint the intestinal barrier regulation linking barrier impairment to inflammation. 

### 4.2. Potential Mechanisms of How a Leaky Gut Leads to Autoimmunity

**Figure 2:**
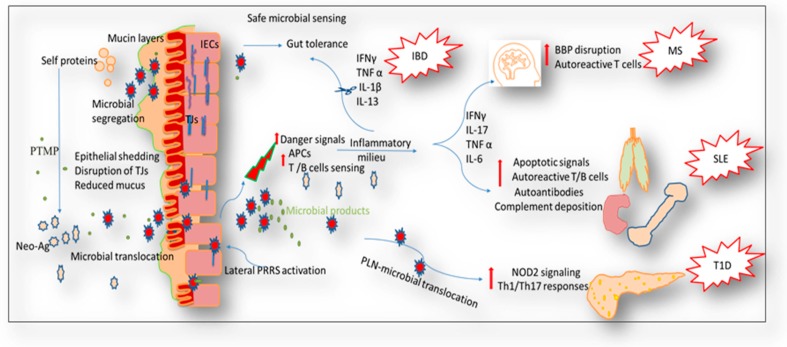
Possible mechanisms of how a leaky gut leads to autoimmunity. Breakdown of the integrity of the intestinal mucosa is associated with microbial dysbiosis. This could result in epigenetic changes of both self-protein and pattern recognition receptor (PRR) sensing. Increased danger signals following microbial dysbiosis provoke immunogenic cell responses, consequently upregulating different pro-inflammatory cytokines levels such as tumor necrosis factor (TNF)α, IFNγ, IL-1, IL-17, IL-6, and IL-13. These inflammatory niches are associated with different pathologies including IBD, MS, SLE, and T1D.

The impaired intestinal barrier and the development of diseases in general have been extensively reviewed [148,168,169]. The paradigm that a leaky gut is a cause or a sequela to autoimmunity is a very active area of research. So far, findings suggest that a leaky gut is a cause of autoimmunity rather than a consequence [130]. In the following section, we will investigate the possible ways by which a leaky gut may contribute to autoimmunity (Figure 2). 

Intestinal microbial dysbiosis is proposed to induce epigenetic modifications that can upregulate TLR expression on APCs [170] and deviate the balance of T-cell subsets [171,172]. Similarly, microbial dysbiosis derives inappropriate post-translational modifications of luminal proteins, creating or exposing neo-antigenic determinants of self-proteins that in turn are recognized by immune cells as autoantigens, thus provoking autoimmunity [173]. Therefore, normalization of microbiota and/or their metabolites may provide a possible therapeutic avenue against autoimmune diseases through regulation of the host epigenome [174]. Moreover, leaky-gut-induced dysbiosis creates an inflammatory environment that can lead to the development of many autoimmune conditions. Intestinal barrier disruption will open the doors for intense immunologic defenses. Recognition of microbes or microbial products by lamina propria defenses will induce escalating proinflammatory cytokines, including IFNγ, TNFα, IL-1β, and IL-13. These cytokines could induce more damage to the intestinal mucosa as observed in chronic IBD [175]. In addition, increased Th1 and Th17 cytokines (IFNγ and IL-17, respectively) as well as enhanced inflammatory activities of macrophages and DCs (e.g., increased IL-6 and TNFα levels) can damage multiple tissues, as observed in SLE. Mechanistically, this is most prominently through the induction of autoreactive T and B cells specific for autoantigens that are a result of the initial tissue destruction and through autoantibody production, with further deposition of immune complexes in tissues which subsequently escalates the damage signals [176]. Similarly, pro-inflammatory cytokines can damage the BBB by breaking down the myelin sheath, which directly contributes to the development of EAE, an experimental rodent model of MS [177]. Furthermore, the breakdown of epithelium associated with a leaky gut may increase microbial translocation to lymphoid tissues, provoking hyperinflammatory responses. For instance, microbial translocation to the pancreatic lymph nodes (PLNs) has been proposed to activate the nucleotide-binding oligomerization domain containing protein 2 (NOD2) signaling, initiating the myeloid proinflammatory cytokines IL-6 and TNFα, thus enhancing the Th1/Th17-induced damage of pancreatic β cells. These events lead to pancreatic insulitis, which is associated with T1D, and can reestablish T1D in a diabetes-resistant mouse model [178].

### 4.3. Involvement of a Leaky Gut in Specific Autoimmune Diseases

Gut dysbiosis is a nidus for the breakdown of immune tolerance at the mucosal interface, as we have discussed. A complex circuit of inflammation that could be manifested in any or all of the above mentioned autoimmune diseases indicates that a leaky gut may be a core for the initiation and progression of these pathologies. Here, we further discuss the involvement of a leaky gut in specific autoimmune conditions.

#### 4.3.1. T1D

Even though the link between a leaky gut and T1D pathogenesis has been questioned [179], an expanding pool of literature has reviewed the link between microbiota dysbiosis and T1D pathogenesis [180,181]. Dysbiotic microbiota could represent a significant contributor to the development of T1D in animals with genetically predisposed backgrounds. For example, similarities in microbiota composition and gut dysfunction were observed among NOD mice housed in different facilities [126], indicating that a leaky gut may be a major player for T1D development. Importantly, impaired microbiota and their metabolites have been reported in human T1D patients, indicating the establishment of a leaky gut [125]. 

Diet-induced modulation of intestinal integrity and microbiota diversity that enhances the *Firmicutes/Bacteroidetes* ratio has been associated with impaired progression of T1D [182]. Additionally, feeding the NOD mice with an acetate/butyrate-producing specialized diet decreased gut permeability and boosted against T1D by diminishing the IL-21 inflammatory diabetogenic niche. Moreover, acetate-yielding diets markedly reduced the proliferation of autoreactive lymphocytes, and butyrate-producing diets upregulated Treg differentiation [183]. Interestingly, the reshaping of acetate-to-butyrate-producing gut microbiota ratios through fecal transfer from T1D-protected mice to germ-free (GF) NOD mice protected against T1D development in GF mice as well [184]. These findings further support the notion that the correction of leaky-gut-derived microbiota dysbiosis can be a therapeutic approach for T1D and other T-cell-mediated autoimmune conditions. Nevertheless, further studies on the gut microbiome–T1D axis are warranted.

#### 4.3.2. MS

Although the exact etiology of this multifactorial neurological disorder is debated, a growing amount of evidence [185] links the onset and progression of MS to gut leakiness [127,128]. It has been proposed that dysbiotic microbiota may trigger MS neuroinflammation directly through their metabolites, which can cross the BBB and affect immune cells within the CNS [186]. However, even though microbiota dysbiosis has been established in many cases of MS, only the idea that MS is inflammatorily driven is widely accepted. The microbiota signature associated with the development of MS is actually speculative. Some argue that there is no typical signature that is equally manifested among different MS patients [187], while others speculate that the consistencies in certain microbiome signatures can be biomarkers for the diagnosis of MS [188].

Indeed, alteration of microbiota and their metabolites could be promising targets for treatment of MS, where the functional gut microbiota could contribute to the health of CNS in general, as previously reviewed [189]. Targeting of microbiota delayed EAE onset and hindered the disease progression in animal models [190]. Similarly, in pilot studies of clinical MS patients, correction of microbial dysbiosis was able to establish an anti-inflammatory niche through enhancing TGFβ/IL-10 levels, reducing IL-17 producing CD4^+^ T cells, and favoring Treg proliferation, which improved disease relapse [191]. Therefore, more studies on the microbiome–brain link may provide novel avenues for the treatment of MS.

#### 4.3.3. IBD

A leaky gut or impaired intestinal permeability could be a signal for IBD initiation [192,193], as many observations have been shown to correlate defective intestinal epithelial barriers to IBD pathogenesis [194]. The interaction between barrier permeability, gut microbiota, and mucosal immune sensing and how this tri-circuit could lead to IBD have been extensively reviewed elsewhere [195,196]. Briefly, disruption of intestinal membrane TJs [164] and breaching of the intestinal mucosa can induce exaggerated responses to the gut microbiota which greatly contribute to IBD pathogenesis in genetically predisposed individuals [197].

Thus, combined therapies that involve immunomodulators [198,199], microbiota targeting [200,201] using commensals such as *Escherichia coli* [202], and probiotics such as *Lactobacilli* spp. [203], as well as normalization of barrier functions [195], might provide some relief for IBD patients. However, with emerging findings indicating that even with response to therapies, many patients still have ongoing progressive disease symptoms and increased intestinal permeability in spite of mucosal healing [204], other aspects of leaky-gut-induced IBD should be considered. For instance, targeting TJ proteins and improving IEC repair and proliferation may be therapeutically worthy, especially with promising findings that suggest a positive feedback loop between normalization of TJs [205] and epithelial modulation [206].

#### 4.3.4. SLE

The relationship between dysbiotic microbiota, for example, the reduced *Firmicutes/Bacteroidetes* ratio, and SLE has been established [207]. Gut dysbiosis and increased intestinal permeability have been reported in both experimental animal models and human patients with lupus [207,208]. Altered microbiota composition was correlated to lupus progression in human patients and an NZB/W F1 lupus mouse model [209]. Recent data from our laboratory showed how microbiota manipulation and reversal of the leaky gut could improve disease prognosis. For instance, normalization of gut microbiota through antibiotics such as vancomycin improved epithelial integrity, as shown by decreased small intestinal and colonic permeability to FITC-conjugated dextran [210]. In addition, vancomycin enhanced the paracellular integrity by upregulating TJ proteins and reducing the translocation of bacteria or their products (e.g., LPS) to the mesenteric lymph nodes (MLN) and systemic circulation [210]. Similarly, correction of the leaky gut in MRL/lpr mice reversed the inflammatory niches and diminished lupus-related damage of extraintestinal organs such as the kidney in female mice [122]. These findings from our team provide further support for the implication of gut leakiness and microbiota dysbiosis in lupus progression and highlight the importance of microbiome-mediated therapy for disease control.

## 5. Potential Roles of RA on the Leaky Gut and, Subsequently, Autoimmune Diseases

Generally, the inverse relationship between serum retinol levels and gut permeability indicates a direct role of vitamin A metabolites in maintaining barrier functionality [211,212]. The data we have gathered and discussed throughout this review direct us to an interesting yet intricate question: Does RA directly improve the intestinal integrity and consequently hinder the autoimmunity, or does it exert indirect effects through regulating the gut microbiota? Notably, similar patterns of dysbiotic gut microbiota has been observed with VAD and clinical cases of autoimmunity. For instance, increased abundance of *Bacteroides* such as *Bacteroides vulgatus*, which has been associated with autoimmune conditions like T1D [213] and MS [214], has been observed in VAD [215]. Thus, here we will discuss several hypotheses that might suggest multiple interacting functions of RA.

### 5.1. RA and TJ Proteins

Impairment of TJs could represent a start for autoimmune pathologies. Therefore, exploring whether a dietary metabolite such as RA improves the expression and/or assembly of TJ proteins may provide novel therapeutic targets against autoimmune diseases.

RA has been reported to modulate the expression of cellular TJ proteins in various host tissues including the skin [216], kidney [217], and peritoneal tissues [218]. RA could affect intestinal permeability directly through the role of its RARγ–RXRα heterodimers in formation of functional TJs of epithelial cells during visceral endoderm differentiation [219]. RA regulates the expression of TJ genes in epithelial cells. For example, RA supplementation to epithelial cells grown in transwell systems induced RARα-mediated transcriptional changes in the expression of different TJ proteins, including Claudin-1, Claudin-4, and ZO1, consequently augmenting the TER of the epithelial monolayer [220]. Furthermore, RA–RARβ signaling has been found to enhance the expression of intestinal membrane proteins such as a sulfate anion transporter known as Down-Regulated in Adenoma (DRA) [221], which could improve TJ expression and counteract DSS-induced colitis [222]. Indirectly, the modulation of TJ gene expression through RA can be linked to the ability of RA to alter TLR-microbial sensing. For example, RA treatment to Caco-2 cells enhanced their TER through RARβ-mediated upregulation of TLR4, which in turn improved the expression of ZO-2, whereas the expression of TLR4 and ZO-2 was improved or diminished by the activation or silencing of RARβ, respectively [161]. TLR4 was a prerequisite for RARβ-induced ZO-2 expression, as RARβ cannot directly bind to the ZO-2 gene promoter [161]. Moreover, RA can counteract oxidative-stress-induced disassembly of TJs. For example, in vitro treatment of RA or an RARα agonist (Am580) could ameliorate the disruption of the epithelial monolayer TJs that had been induced by hydrogen peroxide (H_2_O_2_) [220].

However, RA-directed regulation of TJ proteins is not well established for all tissues. For instance, RA was neither essential nor able to induce the expression of BBB TJs or adherent proteins in brain tissues [223]. This might direct us to our second hypothesis, which is that RA indirectly modulates the leaky gut through targeting the gut microbiota.

### 5.2. Indirect Regulation through the Interaction of RA and Gut Microbiota

The crosstalk between microbial communities and RA could imprint immune cell functions to not only maintain gut homeostasis but also regulate the functionality of peripheral lymphoid organs. For example, gut colonization with commensal fungi induced the migration of LP CD103^+^ RALDH^+^ DCs to the peripheral lymph nodes, where RA production by these migrating cells enhanced the homing capacity of the peripheral lymphocytes to the gut-associated lymphoid tissues and peripheral lymph nodes [224]. Moreover, induction of oral tolerance through microbial superantigens (e.g., staphylococcal enterotoxin A) has been linked to the ability of intestinal DCs to produce RA and the subsequent upregulation of Treg cells both in vivo and in vitro [225]. Furthermore, gut microbiota produce short-chain fatty acids that can also enhance RA production by IECs and contribute to the immunoregulatory functions of IECs through promoting tolerogenic DCs [147]. Those findings highlight a circuit of interaction between gut microbiota and RA signaling. 

Recent findings highlight the link between the RA–microbiome axis and regulation of B-cell responses in the gut. Gut bacteria can produce acetate, and RA signaling is required for acetate-induced programming of intestinal B cells to become IgA-secreting cells [226]. Similarly, RA signaling is correlated with normalized gut microflora composition after the hapten 4-hydroxy-3-nitrophenyl acetyl-cholera toxin (NP-CT) oral immunization. In addition, RA signaling in B cells is essential for their class switching to IgA-secreting cells and consequently their gut immune function. Silencing of RARα signaling was associated with reduced IgA-secreting cells and increased proportions of *Lachnospiraceae* and *Lactobacillus/Streptococcus* in fecal samples [37]. These results associate RA signaling with gut microbiota in the regulation of B-cell responses. 

Furthermore, shaping microbiota composition and their metabolites via RA can derive tolerogenic Treg responses [227]. RA has been found to be essential for inducing the proliferation of Foxp3^+^ T cells and for promoting IL-10 upregulation in vitro [228]. Interestingly, RA signaling in human monocyte-derived (Mo)-DCs and myeloid-DCs is the prerequisite for *B. infantis*-induced immunomodulatory responses [229]. Production of RA by CD103^+^ DCs was directly linked to the enhanced abilities of *B. infantis* to control DDS-induced colitis by reducing both Th1 and Th17 populations*.* However, *B. infantis* maintains its effects on upregulation of Treg cells in the absence of RA signaling, suggesting an RA-independent mechanism as well [229]. Furthermore, an altered microbiota composition, such as reduced segmented filamentous bacteria (SFB), has been linked to abnormal regulation of Th17 cells through a DC-independent mechanisms in the absence of RA [230]. 

Indeed, the capability of RA signaling to shape the responses of mucosal immune cells depends on the specific microbiome microenvironment. For instance, in response to RARα sensing, different commensal and probiotic microbiota may influence the programming of human Mo-DCs and T-cell polarization differently. Stimulating human Mo-DCs with *E. coli* or *Morganella morganii* provoked pronounced pro-inflammatory responses indicated by increased IFNγ-producing T cells and IL-17 responses [231]*.* However, conditioning of Mo-DCs with RA could indirectly shift T-cell programming. RA was able to reduce DCs’ costimulatory signals, leading to mitigation of Th1 activation and minimal or undetectable Th17 responses against *E. coli* and *M. morganii* [231]. 

Results from our laboratory have demonstrated that altered *Lachnospiraceae* and *Lactobacillus* populations were associated with the development of autoimmune pathologies in SLE*.* Restoring such imbalance via RA—specifically, the upregulation of *Lactobacillus* colonization—can improve lupus symptoms [121]. In vitro findings provided more support to the interplay between RA and *Lactobacillus* spp. and the possible modulation of intestinal mucosal immunity. Treatment of mucosal DC cultures with the cell free supernatant of *L. reuteri* has been shown to augment RA-induced tolerogenic responses, such as increased IL-10 and Foxp3 expression, as well as reduced expression of several genes responsible for Ag uptake and presentation [232]. Interestingly, recent data showed the importance of RA–RAR signaling for maintaining gut microbiota homeostasis, where the absence of RARα signaling was associated with overexpression of antimicrobial peptides such as Reg3γ from intestinal Paneth cells. This resulted in microbial dysbiosis represented by altered bacterial colonization with remarkable differences in the abundance of lactobacilli in the colon and SFB in both the ileum and colon of RARα^villin^ mice [145].

Therefore, to determine whether RA directly modulates intestinal permeability and autoimmunity or indirectly through regulating the gut microbiota and specifically *Lactobacillus* spp., we could propose a study where the effects of RA are evaluated in the absence of *Lactobacillus* spp. It is likely that RA exerts protective effects against a leaky gut and, subsequently, autoimmune pathologies through both direct and indirect mechanisms. 

## 6. Concluding Remarks and Future Directions

A significant amount of literature on the gut–autoimmune axis has reported on the involvement of a leaky gut in different autoimmune diseases, including T1D, MS, IBD, and SLE. With the side effects associated with immunosuppressive drugs that are currently used for controlling autoimmune pathologies and increased public awareness, the need for safer immunotherapeutics is imperative. Thus, natural metabolites such as RA may be a less risky approach for dealing with autoimmunity. However, as RA works with a microenvironment-specific mechanism that derives distinct and even controversial consequences, more studies are needed to unravel its immunomodulatory actions under different circumstances. Furthermore, although beyond the scope of our review, evidence exists linking RA toxicity to several acute and chronic diseases [233,234]. There is also evidence that RA can cause a temporary feedback inhibition to the production of endogenous RA itself [235,236] Therefore, many of the short-term observations described in our review may be temporary consequences due to the feedback inhibitory effect of RA and that longer-term observations could actually reveal toxic effects that may, in fact, indicate a causal role for RA in these same autoimmune diseases. Thus, our hypothesis on the link between RA, leaky gut, and autoimmunity is relatively weak and requires more supporting evidence. Dissecting the complex roles of RA in different phases of autoimmune disease progression, understanding the cell-specific roles of RAR signaling, and studying how RA interplays differently with different microbiotas will improve our understanding of the immunoregulatory circuits led by RA. This may bring us closer to controlling different autoimmune pathologies. As RA has many immunomodulatory functions, uncovering more about the underlying cellular and molecular mechanisms behind the RA–microbiome axis is a very interesting area to explore.
